# Dichlorido[2,2′-(1,10-phenanthrolin-2-ylimino)diethanol]cadmium(II)

**DOI:** 10.1107/S1600536809037465

**Published:** 2009-09-19

**Authors:** Shi Guo Zhang, Long Miao Xie

**Affiliations:** a Department of Chemistry and Chemical Engineering, Institute of Materials Chemistry, Binzhou University, Binzhou 256603, People’s Republic of China; b Department of Chemistry, Shandong Normal University, Jinan 250014, People’s Republic of China

## Abstract

In the title complex, [CdCl_2_(C_16_H_17_N_3_O_2_)], the metal atom exhibits a distorted trigonal-bipyramidal coordination geometry. O—H⋯O and O—H⋯Cl hydrogen bonds involving hydr­oxy groups and one of coordinated Cl atoms link complexes in the crystal packing. There is a π–π stacking inter­action between adjacent 1,10-phenanthroline rings, with a distance of 3.675 (2) Å between the centroids of the pyridine and benzene rings.

## Related literature

For related structures, see: Jin & Li (2009 ▶); Zhang *et al.* (2008 ▶).
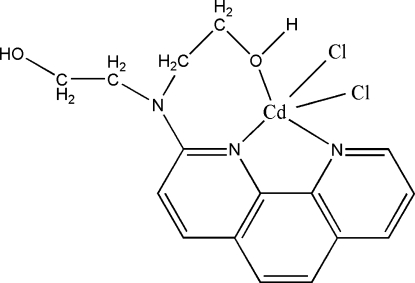


## Experimental

### 

#### Crystal data

[CdCl_2_(C_16_H_17_N_3_O_2_)]
*M* *_r_* = 466.63
Monoclinic, 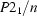

*a* = 7.9435 (15) Å
*b* = 22.548 (4) Å
*c* = 9.5216 (18) Å
β = 98.808 (3)°
*V* = 1685.3 (5) Å^3^
*Z* = 4
Mo *K*α radiation
μ = 1.63 mm^−1^
*T* = 298 K
0.24 × 0.11 × 0.05 mm

#### Data collection

Bruker SMART APEX CCD diffractometer
Absorption correction: multi-scan (*SADABS*; Sheldrick, 1996 ▶) *T*_min_ = 0.696, *T*_max_ = 0.923
9776 measured reflections
3641 independent reflections
2933 reflections with *I* > 2σ(*I*)
*R*_int_ = 0.040

#### Refinement

*R*[*F* ^2^ > 2σ(*F* ^2^)] = 0.039
*wR*(*F* ^2^) = 0.087
*S* = 1.03
3641 reflections
217 parameters
2 restraints
H-atom parameters constrained
Δρ_max_ = 0.54 e Å^−3^
Δρ_min_ = −0.40 e Å^−3^

### 

Data collection: *SMART* (Bruker, 1997 ▶); cell refinement: *SAINT* (Bruker, 1997 ▶); data reduction: *SAINT*; program(s) used to solve structure: *SHELXTL* (Sheldrick, 2008 ▶); program(s) used to refine structure: *SHELXTL*; molecular graphics: *SHELXTL*; software used to prepare material for publication: *SHELXTL* and local programs.

## Supplementary Material

Crystal structure: contains datablocks I, global. DOI: 10.1107/S1600536809037465/kp2226sup1.cif

Structure factors: contains datablocks I. DOI: 10.1107/S1600536809037465/kp2226Isup2.hkl

Additional supplementary materials:  crystallographic information; 3D view; checkCIF report

## Figures and Tables

**Table:** 

Cd1—N3	2.235 (3)
Cd1—O2	2.279 (2)
Cd1—N2	2.470 (3)
Cd1—Cl1	2.4752 (11)
Cd1—Cl2	2.4800 (10)

**Table:** 

N3—Cd1—O2	136.77 (9)
N3—Cd1—N2	72.40 (9)
O2—Cd1—N2	78.60 (9)
N3—Cd1—Cl1	106.83 (7)
O2—Cd1—Cl1	112.94 (7)
N2—Cd1—Cl1	110.02 (7)
N3—Cd1—Cl2	98.90 (8)
O2—Cd1—Cl2	90.69 (7)
N2—Cd1—Cl2	150.32 (7)
Cl1—Cd1—Cl2	99.66 (4)

**Table 2:** Hydrogen-bond geometry (Å, °)

*D*—H⋯*A*	*D*—H	H⋯*A*	*D*⋯*A*	*D*—H⋯*A*
O2—H7⋯O1^i^	0.85	1.84	2.670 (4)	165
O1—H6⋯Cl1^ii^	0.85	2.34	3.157 (3)	162

Symmetry codes: (i) 
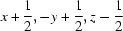
; (ii) 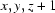.

**Fig. 1.**
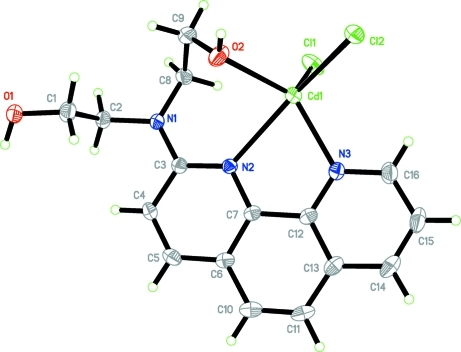
Structure of title complex with the atom-numbering scheme. Displacement ellipsoids are drawn at the 30% probability level.

**Fig. 2.**
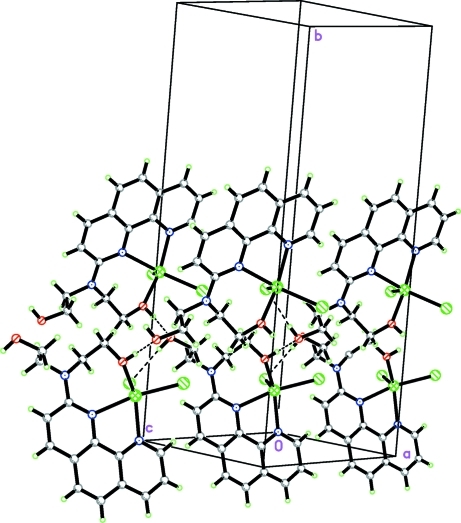
The crystal structure with hydrogen bonds shown as dashed lines.

## supplementary crystallographic information

### Comment

Derivatives of 1,10-phenanthroline play an important role in modern coordination chemistry and a lot of complexes have been published with this type of ligands (see Zhang *et al.*, 2008). Although compound 2,2'-(1,10-phenanthrolin-2-ylimino)diethanol has been published (see Jin *et al.*, 2009) but its complex  has not been available. Herein we report the crystal structure of Cd^II^ complex with 2,2'-(1,10-phenanthrolin-2-ylimino)diethanol as the ligand. The Cd1 atom reveals a distorted trigonal bipyramidal coordination (Fig. 1 and Table 1). O—H···O hydrogen bond between hydroxyl groups and O—H···Cl hydrogen bond connect the complexes (Fig. 2 and Table 2). There is a  π–π stacking interaction involving symmetry-related 1,10-phenanthroline rings, with the relevant distances being *Cg*1···*Cg*2^i^ = 3.675 (2) Å and *Cg*1···*Cg*2^i^_perp_ = 3.509 Å; α is 0.71° [symmetry code: (i) -*x*, -*y*, 1 - *z*; *Cg*1 and *Cg*2 are the centroids of C3—C7/N2 ring and C6C7C10C13 ring, respectively; *Cg*1···*Cg*2_perp_ is the perpendicular distance from ring *Cg*1 to ring *Cg*2^i^; α is the dihedral angle between the *Cg*1 ring plane and the *Cg*2 ring plane].

### Experimental

10 ml methanol solution of 2,2'-(1,10-phenanthrolin-2-ylimino)diethanol (0.0439 g, 0.155 mmol) was added into 10 ml H_2_O solution containing CdCl_2_.2.5 H_2_O (0.0354 g, 0.155 mmol) and the mixture was stirred for a few m. The colourless single crystals were obtained after the filtrate had been allowed to stand at room temperature for two weeks.

### Refinement

HO-bound H atoms were located in a difference Fourier map, and placed in idealised positions with O—H = 0.85 Å and with *U*_iso_(H) = 1.5*U*_eq_(O); other H atoms were placed in calculated positions with C—H = 0.97 Å for methylene group and C—H = 0.93 Å for 1,10-phenanthroline ring with *U*_iso_(H) = 1.2*U*_eq_(C). All H atoms were refined as riding entities.

### Crystal data

**Table:**

[CdCl_2_(C_16_H_17_N_3_O_2_)]	*F*(000) = 928
*M**_r_* = 466.63	*D*_x_ = 1.839 Mg m^−^^3^
Monoclinic, *P*2_1_/*n*	Mo *K*α radiation, λ = 0.71073 Å
Hall symbol: -P 2yn	Cell parameters from 2277 reflections
*a* = 7.9435 (15) Å	θ = 2.4–24.0°
*b* = 22.548 (4) Å	µ = 1.63 mm^−^^1^
*c* = 9.5216 (18) Å	*T* = 298 K
β = 98.808 (3)°	Prism, colourless
*V* = 1685.3 (5) Å^3^	0.24 × 0.11 × 0.05 mm
*Z* = 4	

### Data collection

**Table:**

Bruker SMART APEX CCD diffractometer	3641 independent reflections
Radiation source: fine-focus sealed tube	2933 reflections with *I* > 2σ(*I*)
graphite	*R*_int_ = 0.040
φ and ω scans	θ_max_ = 27.0°, θ_min_ = 1.8°
Absorption correction: multi-scan (*SADABS*; Sheldrick, 1996)	*h* = −10→9
*T*_min_ = 0.696, *T*_max_ = 0.923	*k* = −28→24
9776 measured reflections	*l* = −11→12

### Refinement

**Table:**

Refinement on *F*^2^	Primary atom site location: structure-invariant direct methods
Least-squares matrix: full	Secondary atom site location: difference Fourier map
*R*[*F*^2^ > 2σ(*F*^2^)] = 0.039	Hydrogen site location: inferred from neighbouring sites
*wR*(*F*^2^) = 0.087	H-atom parameters constrained
*S* = 1.03	*w* = 1/[σ^2^(*F*_o_^2^) + (0.0368*P*)^2^] where *P* = (*F*_o_^2^ + 2*F*_c_^2^)/3
3641 reflections	(Δ/σ)_max_ = 0.003
217 parameters	Δρ_max_ = 0.54 e Å^−^^3^
2 restraints	Δρ_min_ = −0.40 e Å^−^^3^

### Special details

**Table:**

Geometry. All e.s.d.'s (except the e.s.d. in the dihedral angle between two l.s. planes) are estimated using the full covariance matrix. The cell e.s.d.'s are taken into account individually in the estimation of e.s.d.'s in distances, angles and torsion angles; correlations between e.s.d.'s in cell parameters are only used when they are defined by crystal symmetry. An approximate (isotropic) treatment of cell e.s.d.'s is used for estimating e.s.d.'s involving l.s. planes.
Refinement. Refinement of *F*^2^ against ALL reflections. The weighted *R*-factor *wR* and goodness of fit *S* are based on *F*^2^, conventional *R*-factors *R* are based on *F*, with *F* set to zero for negative *F*^2^. The threshold expression of *F*^2^ > σ(*F*^2^) is used only for calculating *R*-factors(gt) *etc*. and is not relevant to the choice of reflections for refinement. *R*-factors based on *F*^2^ are statistically about twice as large as those based on *F*, and *R*- factors based on ALL data will be even larger.

### Fractional atomic coordinates and isotropic or equivalent isotropic displacement parameters (Å^2^)

**Table:**

	*x*	*y*	*z*	*U*_iso_*/*U*_eq_	
C1	0.1374 (5)	0.21631 (17)	0.8042 (4)	0.0422 (9)	
H1A	0.2248	0.1869	0.8330	0.051*	
H1B	0.1863	0.2473	0.7526	0.051*	
C2	−0.0096 (5)	0.18806 (15)	0.7096 (3)	0.0349 (8)	
H2A	−0.0637	0.1594	0.7641	0.042*	
H2B	−0.0931	0.2183	0.6761	0.042*	
C3	0.1122 (4)	0.10255 (14)	0.5974 (3)	0.0289 (7)	
C4	0.1161 (5)	0.07105 (15)	0.7281 (4)	0.0368 (9)	
H4	0.0798	0.0894	0.8057	0.044*	
C5	0.1724 (5)	0.01476 (16)	0.7386 (4)	0.0386 (9)	
H5	0.1720	−0.0059	0.8231	0.046*	
C6	0.2315 (4)	−0.01327 (14)	0.6243 (4)	0.0316 (8)	
C7	0.2289 (4)	0.02028 (14)	0.4999 (3)	0.0272 (7)	
C8	0.0074 (5)	0.19041 (15)	0.4534 (4)	0.0386 (9)	
H8A	−0.0969	0.2128	0.4539	0.046*	
H8B	−0.0132	0.1620	0.3761	0.046*	
C9	0.1446 (5)	0.23235 (16)	0.4243 (4)	0.0483 (10)	
H9A	0.1064	0.2550	0.3388	0.058*	
H9B	0.1724	0.2598	0.5030	0.058*	
C10	0.2929 (5)	−0.07252 (15)	0.6327 (4)	0.0418 (9)	
H10	0.2950	−0.0934	0.7172	0.050*	
C11	0.3475 (5)	−0.09907 (16)	0.5234 (4)	0.0426 (10)	
H11	0.3859	−0.1381	0.5319	0.051*	
C12	0.2908 (4)	−0.00865 (14)	0.3824 (4)	0.0298 (8)	
C13	0.3472 (4)	−0.06791 (14)	0.3938 (4)	0.0351 (8)	
C14	0.4001 (5)	−0.09367 (17)	0.2731 (5)	0.0476 (10)	
H14	0.4379	−0.1327	0.2769	0.057*	
C15	0.3964 (5)	−0.06231 (18)	0.1520 (5)	0.0523 (11)	
H15	0.4297	−0.0796	0.0719	0.063*	
C16	0.3424 (5)	−0.00404 (17)	0.1491 (4)	0.0452 (10)	
H16	0.3409	0.0176	0.0658	0.054*	
Cd1	0.22860 (3)	0.118861 (11)	0.25829 (3)	0.03483 (11)	
Cl1	−0.04098 (14)	0.13138 (5)	0.09231 (11)	0.0546 (3)	
Cl2	0.43311 (13)	0.15483 (4)	0.10510 (10)	0.0440 (2)	
N1	0.0451 (4)	0.15814 (11)	0.5862 (3)	0.0301 (6)	
N2	0.1698 (3)	0.07740 (11)	0.4860 (3)	0.0267 (6)	
N3	0.2925 (4)	0.02232 (13)	0.2602 (3)	0.0335 (7)	
O1	0.0806 (3)	0.24096 (10)	0.9274 (2)	0.0452 (7)	
H6	0.0645	0.2146	0.9874	0.068*	
O2	0.2902 (3)	0.19779 (11)	0.4069 (3)	0.0488 (7)	
H7	0.3766	0.2187	0.3977	0.073*	

### Atomic displacement parameters (Å^2^)

**Table:**

	*U*^11^	*U*^22^	*U*^33^	*U*^12^	*U*^13^	*U*^23^
C1	0.045 (2)	0.042 (2)	0.038 (2)	0.0009 (18)	0.0019 (18)	−0.0076 (17)
C2	0.037 (2)	0.037 (2)	0.0319 (19)	0.0038 (16)	0.0070 (16)	−0.0047 (15)
C3	0.0278 (18)	0.0306 (18)	0.0274 (18)	−0.0018 (14)	0.0010 (14)	−0.0002 (14)
C4	0.047 (2)	0.038 (2)	0.0260 (18)	−0.0003 (17)	0.0101 (16)	−0.0003 (15)
C5	0.043 (2)	0.041 (2)	0.031 (2)	−0.0045 (18)	0.0050 (16)	0.0087 (16)
C6	0.0280 (19)	0.0268 (18)	0.037 (2)	−0.0029 (14)	−0.0034 (15)	0.0027 (14)
C7	0.0239 (18)	0.0242 (17)	0.0321 (18)	−0.0029 (14)	0.0001 (14)	0.0000 (13)
C8	0.045 (2)	0.037 (2)	0.034 (2)	0.0105 (17)	0.0083 (17)	0.0059 (16)
C9	0.068 (3)	0.034 (2)	0.047 (2)	0.010 (2)	0.022 (2)	0.0033 (17)
C10	0.037 (2)	0.034 (2)	0.052 (2)	0.0017 (17)	0.0003 (19)	0.0122 (17)
C11	0.037 (2)	0.0249 (19)	0.064 (3)	0.0020 (16)	0.003 (2)	0.0088 (18)
C12	0.0255 (18)	0.0251 (17)	0.037 (2)	−0.0037 (14)	−0.0007 (15)	−0.0024 (14)
C13	0.0260 (19)	0.0278 (19)	0.051 (2)	−0.0035 (15)	0.0043 (16)	−0.0070 (16)
C14	0.042 (2)	0.031 (2)	0.071 (3)	−0.0002 (18)	0.013 (2)	−0.016 (2)
C15	0.058 (3)	0.048 (3)	0.055 (3)	−0.006 (2)	0.025 (2)	−0.023 (2)
C16	0.056 (3)	0.045 (2)	0.037 (2)	−0.005 (2)	0.0134 (19)	−0.0097 (17)
Cd1	0.04436 (19)	0.03078 (16)	0.03027 (16)	0.00004 (12)	0.00864 (12)	0.00178 (11)
Cl1	0.0468 (6)	0.0775 (8)	0.0375 (6)	−0.0090 (5)	0.0002 (5)	0.0155 (5)
Cl2	0.0523 (6)	0.0405 (5)	0.0431 (5)	0.0007 (4)	0.0191 (5)	0.0054 (4)
N1	0.0351 (17)	0.0281 (15)	0.0280 (15)	0.0048 (12)	0.0076 (12)	−0.0010 (12)
N2	0.0291 (16)	0.0244 (14)	0.0262 (15)	−0.0020 (12)	0.0026 (12)	0.0003 (11)
N3	0.0387 (17)	0.0314 (16)	0.0307 (16)	−0.0009 (13)	0.0061 (13)	−0.0043 (12)
O1	0.0655 (19)	0.0362 (14)	0.0326 (15)	0.0092 (13)	0.0034 (13)	−0.0036 (11)
O2	0.0503 (17)	0.0341 (14)	0.0693 (19)	−0.0079 (13)	0.0323 (14)	−0.0100 (13)

### Geometric parameters (Å, °)

**Table:**

C1—O1	1.431 (4)	C9—H9A	0.9700
C1—C2	1.503 (5)	C9—H9B	0.9700
C1—H1A	0.9700	C10—C11	1.329 (5)
C1—H1B	0.9700	C10—H10	0.9300
C2—N1	1.476 (4)	C11—C13	1.420 (5)
C2—H2A	0.9700	C11—H11	0.9300
C2—H2B	0.9700	C12—N3	1.359 (4)
C3—N2	1.344 (4)	C12—C13	1.408 (4)
C3—N1	1.360 (4)	C13—C14	1.408 (5)
C3—C4	1.429 (4)	C14—C15	1.349 (6)
C4—C5	1.344 (5)	C14—H14	0.9300
C4—H4	0.9300	C15—C16	1.381 (5)
C5—C6	1.400 (5)	C15—H15	0.9300
C5—H5	0.9300	C16—N3	1.325 (4)
C6—C7	1.403 (4)	C16—H16	0.9300
C6—C10	1.420 (4)	Cd1—N3	2.235 (3)
C7—N2	1.370 (4)	Cd1—O2	2.279 (2)
C7—C12	1.445 (5)	Cd1—N2	2.470 (3)
C8—N1	1.450 (4)	Cd1—Cl1	2.4752 (11)
C8—C9	1.500 (5)	Cd1—Cl2	2.4800 (10)
C8—H8A	0.9700	O1—H6	0.8482
C8—H8B	0.9700	O2—H7	0.8481
C9—O2	1.425 (4)		
			
O1—C1—C2	110.0 (3)	C10—C11—C13	120.0 (3)
O1—C1—H1A	109.7	C10—C11—H11	120.0
C2—C1—H1A	109.7	C13—C11—H11	120.0
O1—C1—H1B	109.7	N3—C12—C13	120.6 (3)
C2—C1—H1B	109.7	N3—C12—C7	118.7 (3)
H1A—C1—H1B	108.2	C13—C12—C7	120.7 (3)
N1—C2—C1	112.0 (3)	C14—C13—C12	117.4 (3)
N1—C2—H2A	109.2	C14—C13—C11	123.0 (3)
C1—C2—H2A	109.2	C12—C13—C11	119.6 (3)
N1—C2—H2B	109.2	C15—C14—C13	120.7 (4)
C1—C2—H2B	109.2	C15—C14—H14	119.6
H2A—C2—H2B	107.9	C13—C14—H14	119.6
N2—C3—N1	120.2 (3)	C14—C15—C16	118.8 (4)
N2—C3—C4	120.9 (3)	C14—C15—H15	120.6
N1—C3—C4	118.8 (3)	C16—C15—H15	120.6
C5—C4—C3	119.7 (3)	N3—C16—C15	122.8 (4)
C5—C4—H4	120.1	N3—C16—H16	118.6
C3—C4—H4	120.1	C15—C16—H16	118.6
C4—C5—C6	121.1 (3)	N3—Cd1—O2	136.77 (9)
C4—C5—H5	119.5	N3—Cd1—N2	72.40 (9)
C6—C5—H5	119.5	O2—Cd1—N2	78.60 (9)
C5—C6—C7	116.7 (3)	N3—Cd1—Cl1	106.83 (7)
C5—C6—C10	122.3 (3)	O2—Cd1—Cl1	112.94 (7)
C7—C6—C10	121.0 (3)	N2—Cd1—Cl1	110.02 (7)
N2—C7—C6	123.3 (3)	N3—Cd1—Cl2	98.90 (8)
N2—C7—C12	120.1 (3)	O2—Cd1—Cl2	90.69 (7)
C6—C7—C12	116.6 (3)	N2—Cd1—Cl2	150.32 (7)
N1—C8—C9	114.7 (3)	Cl1—Cd1—Cl2	99.66 (4)
N1—C8—H8A	108.6	C3—N1—C8	123.9 (3)
C9—C8—H8A	108.6	C3—N1—C2	121.2 (3)
N1—C8—H8B	108.6	C8—N1—C2	114.7 (3)
C9—C8—H8B	108.6	C3—N2—C7	118.2 (3)
H8A—C8—H8B	107.6	C3—N2—Cd1	131.8 (2)
O2—C9—C8	107.6 (3)	C7—N2—Cd1	109.5 (2)
O2—C9—H9A	110.2	C16—N3—C12	119.7 (3)
C8—C9—H9A	110.2	C16—N3—Cd1	121.7 (3)
O2—C9—H9B	110.2	C12—N3—Cd1	118.4 (2)
C8—C9—H9B	110.2	C1—O1—H6	112.3
H9A—C9—H9B	108.5	C9—O2—Cd1	113.5 (2)
C11—C10—C6	122.0 (4)	C9—O2—H7	113.0
C11—C10—H10	119.0	Cd1—O2—H7	118.1
C6—C10—H10	119.0		
			
O1—C1—C2—N1	−175.8 (3)	N1—C3—N2—C7	−176.9 (3)
N2—C3—C4—C5	−2.4 (5)	C4—C3—N2—C7	1.4 (5)
N1—C3—C4—C5	176.0 (3)	N1—C3—N2—Cd1	12.5 (5)
C3—C4—C5—C6	1.6 (5)	C4—C3—N2—Cd1	−169.2 (2)
C4—C5—C6—C7	0.1 (5)	C6—C7—N2—C3	0.3 (5)
C4—C5—C6—C10	179.5 (3)	C12—C7—N2—C3	179.5 (3)
C5—C6—C7—N2	−1.0 (5)	C6—C7—N2—Cd1	172.9 (2)
C10—C6—C7—N2	179.5 (3)	C12—C7—N2—Cd1	−7.9 (3)
C5—C6—C7—C12	179.7 (3)	N3—Cd1—N2—C3	179.4 (3)
C10—C6—C7—C12	0.2 (5)	O2—Cd1—N2—C3	31.8 (3)
N1—C8—C9—O2	65.3 (4)	Cl1—Cd1—N2—C3	−78.6 (3)
C5—C6—C10—C11	179.4 (4)	Cl2—Cd1—N2—C3	102.6 (3)
C7—C6—C10—C11	−1.2 (5)	N3—Cd1—N2—C7	8.15 (19)
C6—C10—C11—C13	0.6 (6)	O2—Cd1—N2—C7	−139.4 (2)
N2—C7—C12—N3	1.4 (5)	Cl1—Cd1—N2—C7	110.14 (19)
C6—C7—C12—N3	−179.3 (3)	Cl2—Cd1—N2—C7	−68.6 (2)
N2—C7—C12—C13	−178.1 (3)	C15—C16—N3—C12	−1.1 (6)
C6—C7—C12—C13	1.2 (5)	C15—C16—N3—Cd1	174.3 (3)
N3—C12—C13—C14	−1.7 (5)	C13—C12—N3—C16	2.2 (5)
C7—C12—C13—C14	177.8 (3)	C7—C12—N3—C16	−177.3 (3)
N3—C12—C13—C11	178.7 (3)	C13—C12—N3—Cd1	−173.3 (2)
C7—C12—C13—C11	−1.7 (5)	C7—C12—N3—Cd1	7.1 (4)
C10—C11—C13—C14	−178.7 (4)	O2—Cd1—N3—C16	−133.4 (3)
C10—C11—C13—C12	0.8 (5)	N2—Cd1—N3—C16	176.4 (3)
C12—C13—C14—C15	0.1 (5)	Cl1—Cd1—N3—C16	70.2 (3)
C11—C13—C14—C15	179.6 (4)	Cl2—Cd1—N3—C16	−32.8 (3)
C13—C14—C15—C16	1.0 (6)	O2—Cd1—N3—C12	42.1 (3)
C14—C15—C16—N3	−0.6 (6)	N2—Cd1—N3—C12	−8.1 (2)
N2—C3—N1—C8	9.4 (5)	Cl1—Cd1—N3—C12	−114.3 (2)
C4—C3—N1—C8	−169.0 (3)	Cl2—Cd1—N3—C12	142.7 (2)
N2—C3—N1—C2	−176.3 (3)	C8—C9—O2—Cd1	47.5 (3)
C4—C3—N1—C2	5.3 (5)	N3—Cd1—O2—C9	−133.1 (2)
C9—C8—N1—C3	−95.7 (4)	N2—Cd1—O2—C9	−84.7 (2)
C9—C8—N1—C2	89.6 (4)	Cl1—Cd1—O2—C9	22.3 (2)
C1—C2—N1—C3	82.0 (4)	Cl2—Cd1—O2—C9	123.1 (2)
C1—C2—N1—C8	−103.1 (3)		

### Hydrogen-bond geometry (Å, °)

**Table:**

*D*—H···*A*	*D*—H	H···*A*	*D*···*A*	*D*—H···*A*
O2—H7···O1^i^	0.85	1.84	2.670 (4)	165
O1—H6···Cl1^ii^	0.85	2.34	3.157 (3)	162

Symmetry codes: (i) *x*+1/2, −*y*+1/2, *z*−1/2; (ii) *x*, *y*, *z*+1.
